# The Brain Response to Reflectional Symmetry Is Not Uniquely Preattentive

**DOI:** 10.1111/ejn.70608

**Published:** 2026-06-29

**Authors:** Ned Buckley, Alexis D. J. Makin

**Affiliations:** ^1^ Department of Psychological Sciences, Eleanor Rathbone Building University of Liverpool Liverpool UK

**Keywords:** EEG, ERPs, glass patterns, reflection, sustained posterior negativity, symmetry

## Abstract

EEG can be used to measure the brain response to visual regularity. Reflectional symmetry generates an event‐related potential (ERP) named the sustained posterior negativity (SPN). The SPN for reflectional symmetry is generated automatically, whatever the participant's task. This may be because reflectional symmetry has a fundamental role in perceptual organization and guiding adaptive behaviour. In contrast, other types of regularity, such as glass patterns, do not have this ecological significance. We thus predicted that the glass pattern SPN would be more susceptible to experimental variations of task than the reflection SPN. To test this prediction, we ran three experiments on three different groups of 52 participants. All participants saw the same random, reflection and glass dot dipole stimuli. The stimuli were either black or white. In the *Regularity Task*, participants discriminated whether the patterns were random or regular (where regular means reflection or glass). In the *Luminance task*, participants discriminated whether the patterns were black or white. In the *Cross task*, participants discriminated whether the vertical or horizontal arm of the central fixation cross was longer. As predicted, both the reflection and glass SPN were largest in the Regularity task, reduced in the Luminance task and reduced again in the Cross task. Contrary to predictions, glass pattern SPNs were less affected by task than reflection SPNs. This unexpected result suggests that glass patterns may even be processed more automatically than reflection, although this would require replication before it is treated as secure knowledge.

## Graphical abstract

An example of a ‘glass pattern’ used in the current study. The current study investigates brain responses to this type of Regularity across three different tasks and compares it to the brain response to reflectional symmetry patterns.

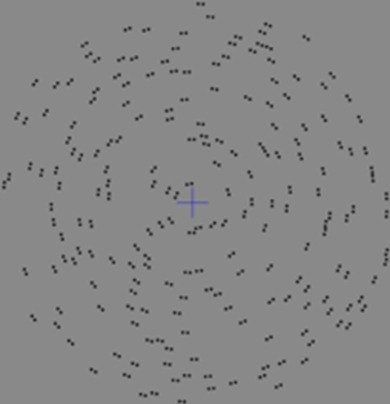

**Abbreviations**
EEG: electroencephalography
ERP: event‐related potential
fMRI: functional magnetic resonance imaging
SPN: sustained posterior negativity

## 1 Introduction

Reflectional symmetry (henceforth reflection) is an ever‐present feature of both the natural and manmade world. For instance, human faces and aeroplanes have an axis of reflection. The left side is a mirror image of the right. Reflection is detected efficiently by human observers, within a single fixation (Barlow and Reeves 1979). Other species also show sensitivity to reflection, with examples in pigeons (Delius and Nowak 1982), bees (Benard et al. 2006) and dolphins (Von Fersen et al. 1992). Many species use reflection to guide adaptive behaviours such as mate and food selection (Møller and Thornhill 1997). Phenotypic reflection is associated with genetic quality and developmental stability, and more symmetrical faces are often judged as more attractive (Grammer et al. 2003; Grammer and Thornhill 1994; Møller and Thornhill 1998). This could explain why sensitivity to reflection is seemingly innate and evident in infants (Bornstein et al. 1981; Bornstein et al. 2023).

**FIGURE 1:**
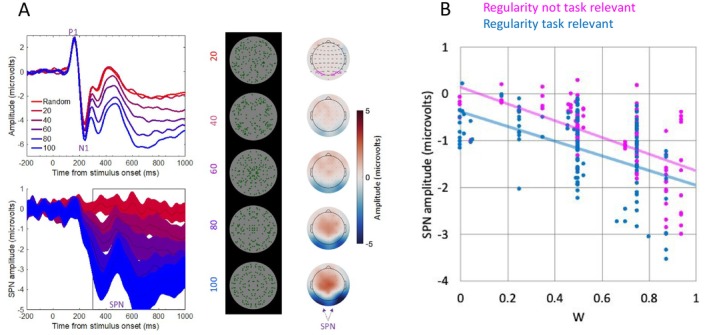
(A) Parametric response to symmetry from Makin et al. (2020). ERPs from the posterior electrode cluster (PO7, O1, O2 and PO8) are shown in the top panel, and difference waves with 95% confidence interval (CI) ribbons are shown in the bottom panel. By convention, a large SPN is one that falls a long way below zero. On the right, topographic difference plots aligned with example stimuli are shown. This highlights SPN amplitude as blue at posterior electrode clusters. (B) Scatterplot of SPNs in The complete Liverpool SPN catalogue. SPN amplitude increases with perceptual goodness (W). The SPN is larger when regularity is task‐relevant (blue dots) than when it is not (pink dots). A similar figure has been used to illustrate the SPN in other recent papers (including Makin et al. 2024). Published under CCBY 4.0 Licence.

Many visual perception studies have demonstrated that reflection is important for perceptual organization (Wagemans et al. 2012), figure‐ground segregation (Driver et al. 1992; Marshall and Halligan 1994) and object recognition (Treder and Meulenbroek 2010). Neuroimaging studies have examined the brain response to reflection. Work using fMRI has shown that early visual areas (V1 and V2) are not activated by reflection. However, many parts of the extrastriate cortex are activated by reflection (Sasaki et al. 2005; Tyler et al. 2005; Kohler et al. 2016; Keefe et al. 2018; Van Meel et al. 2019; Zamboni et al. 2024). This activation can also be measured with electroencephalography (EEG). Most EEG work has used an event‐related potential (ERP) named the ‘Sustained Posterior Negativity’ (SPN). Both random and symmetrical patterns produce ERPs at posterior electrodes, but amplitude is lower for symmetrical patterns from around 200 ms poststimulus onset. The asymmetry–symmetry difference is the SPN. This ERP was first reported by Jacobsen and Höfel (2003) and later refined by Höfel and Jacobsen (2007). A textbook SPN result is illustrated in Figure 1A. A large SPN is one that falls a long way below zero. The more symmetry that is in the stimulus, the larger (i.e., more negative) the SPN (Makin et al. 2020).

All published and unpublished SPN datasets are available in ‘The complete Liverpool SPN catalogue’ (Makin et al. 2022). Based on these projects, one can be confident that the SPN is generated preattentively and automatically, irrespective of the task given to the participants. Although the SPN is larger when participants attend to regularity, it is still present when participants attend to other features of stimuli, such as luminance of the elements (Makin et al. 2024; Buckley and Makin 2024).

One can also be confident that the SPN scales with perceptual goodness. This term refers to subjective salience of a configuration. Although perceptual goodness is subjective, it may be lawfully linked to objective geometrical features. For instance, Van der Helm and Leeuwenberg (1996) proposed the formula *W = E/N*. This considers the ‘holographic identities’ that constitute a given regularity (E) and the total visual information within the regularity (N). Perceptual goodness (W) is easily calculated for dot patterns. For example, for reflection, N is the number of dots, and E is the number of parallel dot pairs with a midpoint falling within the axis of reflection. This means that W = 0.5 and remains consistent with changing numbers of dots (and pairs). Analysis of multiple datasets from the complete Liverpool SPN catalogue indicates W explains substantial variance in SPN amplitude, both when regularity is task‐relevant and when it is not (Figure 1B).

**FIGURE 2:**
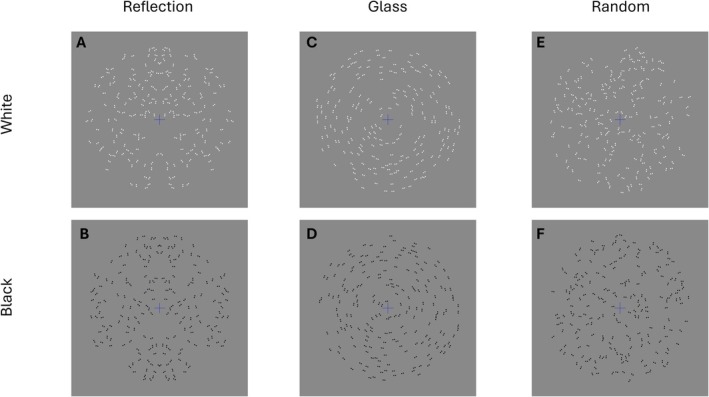
Stimuli used in the current study. Columns show regularity type, and rows show luminance.

Most SPN research has used reflectional stimuli. In contrast, Rampone and Makin (2020) explored SPNs generated by another set of visual regularities known as ‘glass patterns’. Glass patterns are moirés formed by identical dipoles (pairs of dots) that are randomly positioned but coherently oriented according to specific geometric transformations (Glass 1969). The locally paired dots in glass patterns cue the integration of orientation information, meaning these patterns convey the percept of global form. Across different stimulus configurations, spatial frequency and local stimulus statistics remain consistent. According to the formula outlined by Van der Helm and Leeuwenberg (1996), the W score for glass patterns = N dipoles ‐ 1/2N dipoles, and thus it approaches 0.5 when there are many dipoles. Rampone and Makin (2020) compared three different glass patterns: circular, radial and translational. All had a very similar W score to reflection (0.4975 vs. 0.5). Rampone and Makin (2020) found that circular glass patterns produced a comparable SPN amplitude to reflection (circular glass mean SPN amplitude = −2.08 μV, reflection mean SPN amplitude = −2.18 μV). Related results were also found in Makin et al. (2016), Pei et al. (2005), Lestou et al. (2014) and Tyson‐Carr et al. (2022). Circular glass patterns (used in this study) are shown alongside reflection and random dipole arrangements in Figure 2.

Glass patterns are an important class of stimuli, because they have essentially the same perceptual goodness as reflection, but they do not share the same ecological importance as reflection. They are not a property of faces and do not aid figure‐ground organization. Therefore, glass patterns may not be processed automatically and preattentively in the same way as reflection.

It could be that reflection and glass patterns generate the same SPN when participants attend to regularity, but the glass pattern SPN is more fragile to task manipulations than the reflection SPN.

### 1.1 Current Study

The current study tested the prediction that reflection and glass SPNs are differentially sensitive to task. Participants were allocated to one of three tasks. These tasks were the same as some used in Makin et al. (2024). The *Regularity task* required participants to discriminate whether patterns were ‘random’ or ‘regular’ (regular means either reflection or glass). In the *Luminance task*, participants discriminated whether dots were ‘white’ or ‘black’. In the *Cross task*, participants discriminated whether the ‘horizontal’ or ‘vertical’ arm of the central fixation cross was longer. Makin et al. (2024) found that the reflection SPN in the Regularity task was −2.42 μV. This was reduced to −0.84 μV in the Luminance task and reduced even further to −0.72 μV in the Cross task.

It was predicted that the detrimental effect of task would be replicated in the current study for reflection SPNs. It was predicted that the detrimental effect of Task would be even more pronounced for glass SPNs.

Three specific predictions were preregistered:

1. The reflection SPN will be reduced in the Luminance and Cross tasks compared to the Regularity task.
2. The glass SPN will be even more reduced in the Luminance task and Cross tasks compared to the Regularity task.
3. There will be no glass SPN in the Cross task.

## 2 Methods

### 2.1 Participants

A total of 156 participants were recruited. There were 52 participants in the Regularity task (mean age = 20.02, range = 18–43, 6 males, 9 left‐handed), 52 in the Luminance task (mean age = 20.71, range = 18–61, 12 males, 12 left‐handed) and 52 in the Cross task (mean age = 21.08, range = 18–68, 20 males, 5 left‐handed). All had normal or corrected to normal vision. The study was conducted in accordance with the Declaration of Helsinki (Revised 2008), and the protocol was approved by the University of Liverpool Ethics Committee (Approval Code: Ref 13550, approved 31 January 2024).

### 2.2 Power Analysis

The current project had 156 participants with 52 participants completing each of the three tasks. This sample size was preregistered and justified in two ways.

First, the current study needed to confirm apparent pairwise differences between reflection and glass SPN amplitudes with two‐tailed paired samples *t*‐tests. Brysbaert (2019) suggests a typical Cohen's d_z_ is 0.4 in psychology, and 52 participants are required for power to reach 0.8.

The second justification came from an ANOVA power simulation (https://shiny.ieis.tue.nl/anova_power/). The study must be powered to confirm the predicted interaction between stimulus and task with mixed ANOVA. This power simulation was loaded with means [−2, −2; −0.8, −0.5; −0.7 and 0] and SDs of [1.2, 1.2; 1, 0.8; 0.9, 0.5] in the 3 Task × 2 Regularity cells. There were 52 participants in each task. The correlation between within‐subject factors was set to the default *r* = 0.5. The number of virtual samples taken from this virtual population was set to 2000. A study is adequately powered if more than 1600/2000 (80%) of the ANOVAs run on the simulated data yield a significant interaction.

These parameters were justified as follows. Estimated means for the Regularity discrimination task were taken from Rampone and Makin (2020). Estimated means for the Luminance and Cross tasks were taken from Makin et al. (2024). It was assumed that the SDs would be lower than in Rampone and Makin (2020) because of an increased number of trials and shorter stimulus presentation duration. It was also assumed that the SD of amplitudes would scale with the mean amplitude, as this is seemingly universal feature of SPNs (Makin et al. 2022). In Rampone and Makin (2020), there was a large correlation between reflection and glass SPNs (*r* = 0.735). This could be lower in other tasks, so a default correlation of 0.5 for within subjects' variables was employed instead.

With 52 participants in each task, the ANOVA power simulation found 90% power for the Task × Stimulus type interaction, and > 99% power for each main effect.

### 2.3 Apparatus

The EEG experiment used the BioSemi Active‐Two system (Amsterdam, the Netherlands). EEG data were recorded continuously from 64 scalp electrodes arranged according to the extended international 10–20 system. Bipolar VEOG and HEOG external channels were used to monitor for ocular artefacts but were not included in ERP analysis. The participant's head position was stabilized with a chin rest positioned 57 cm from a 51 × 29 cm (1920 × 1080 pixel) HP E233 LED backlit monitor with a 60‐Hz refresh rate. The experiment was conducted in a darkened and electrically shielded room. Experimental programming was completed in Python using open‐source PsychoPy3 software (Peirce 2007).

### 2.4 Stimuli

Stimuli examples can be seen in Figure 2. Stimuli generation was the same as in Rampone and Makin (2020), but with two exceptions. Firstly, only three types of patterns were generated for the current study (circular glass, reflection and random), and secondly, stimuli in the current study displayed white dot dipoles in half the trials and black dot dipoles in the other half. Dipoles were made with two dots with radius 0.04°. The radius of a dipole (distance from the centre of a dot and the centre of the dipole) was 0.08°. Dipole locations were restricted within the circumference of an outer circular region with radius 6.4° and an inner circular region, around the central fixation point, with radius 0.5°. The minimum distance between dipoles was 0.26°. All patterns were made of 200 dipoles (100 dipoles on each side of the central vertical meridian). W‐score for glass patterns was W = 199/400 = 0.4975, whereas W for reflection was W = 100/200 = 0.5.

### 2.5 Design

The current study employs a mixed design. All participants viewed the same six types of stimuli: 3 Regularity (reflection, glass and random) × 2 Luminance (black and white). They were assigned to one of the three tasks (Regularity, Luminance or Cross). Stimuli were presented in a randomized order in each task. Each task involved a total of 640 trials (320 random, 160 reflection and 160 glass).

### 2.6 Procedure

**FIGURE 3:**
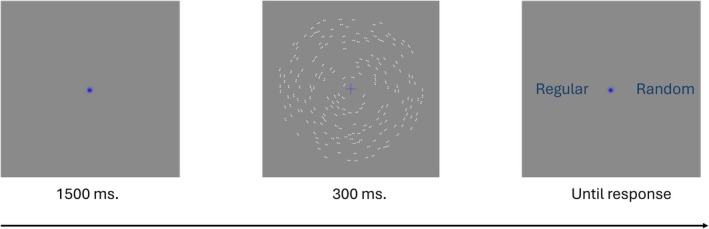
Trial structure of the current study. Each trial began with a 1500‐ms baseline, followed by stimulus presentation for 300 ms. Participants are then shown the response screen until a response is recorded. The example shown here is from the Regularity task, so participants answer whether they deem the pattern ‘Regular’ or ‘Random’. In the Luminance task response, options read ‘White’ and ‘Black’, in the Cross task, they read ‘Vertical’ and ‘Horizontal’. Response mapping was randomized for every trial in all three tasks.

Each trial began with a 1500‐ms baseline, followed by stimulus presentation for 300 ms (Figure 3). Following stimulus offset, participants classified patterns as ‘Regular’ or ‘Random’ (Regularity task) or ‘White’ or ‘Black’ (Luminance task). In the Cross task, participants answered with which arm of the central fixation cross was longer, either ‘Vertical’ or ‘Horizontal’. Responses were entered using the left (A) and right (L) keys. Response mapping was randomized to prevent lateralized motor preparation during the presentation interval. The word ‘wrong’ appeared in red for 1500 ms if participants entered an incorrect response. A practice block of 16 trials preceded each task.

### 2.7 EEG Processing

The processing pipeline was the same as that in Buckley and Makin (2024). EEG data were analysed using EEGLAB 2022.1 functions in MATLAB 2023a. All raw data and analysis scripts are available in the Project 49 folders on ‘The complete Liverpool SPN catalogue’.

EEG datasets were first re‐referenced to the scalp average, low‐pass filtered at 25 Hz and downsampled to 256 Hz. Continuous data were then segmented into −500 to +500 ms epochs with a −200 ms prestimulus baseline. Noisy channels were identified and zeroed during artefact rejection using independent components analysis (ICA). Components capturing large artefacts were identified and removed using the adjust procedure (Regularity task: min = 2, max = 18, average = 6.81; Luminance task: min = 1, max = 18, average = 6.67; Cross task: min = 1, max = 20, average = 7.31). After this, noisy channels were replaced using spherical interpolation (Regularity task: 24 participants had at least 1 channel interpolated; Luminance task: 25 participants had at least 1 channel interpolated; Cross task: 23 participants had at least 1 channel interpolated). Data were then averaged over remaining trials for each condition for each participant.

### 2.8 Analysis

The SPN was defined as the symmetry–asymmetry amplitude difference averaged across the posterior electrode cluster [PO7, O1, O2 and PO8]. This was completed across the 250–400‐ms time window for the Regularity and Luminance tasks, and across the 250–300‐ms window for the Cross task. The unmatched time windows were justified by Makin et al. (2024), and the spatiotemporal cluster was preregistered. SPN amplitude was then analysed in a mixed ANOVA including the within‐subject factor of stimulus (reflection and glass) and the between‐subjects factor of task (Regularity, Luminance or Cross).

## 3 Results

### 3.1 Behavioural Results

Participants found the Luminance task easiest (min correct = 89.06%, max correct = 99.84%, average = 97.95%). The Regularity task was also relatively easy (min correct = 73.13%, max correct = 98.59%, average correct = 90.48%). The Cross task was the hardest (min correct = 52.97%, max correct = 94.69%, average = 80.74%).

### 3.2 ERP Results

**FIGURE 4:**
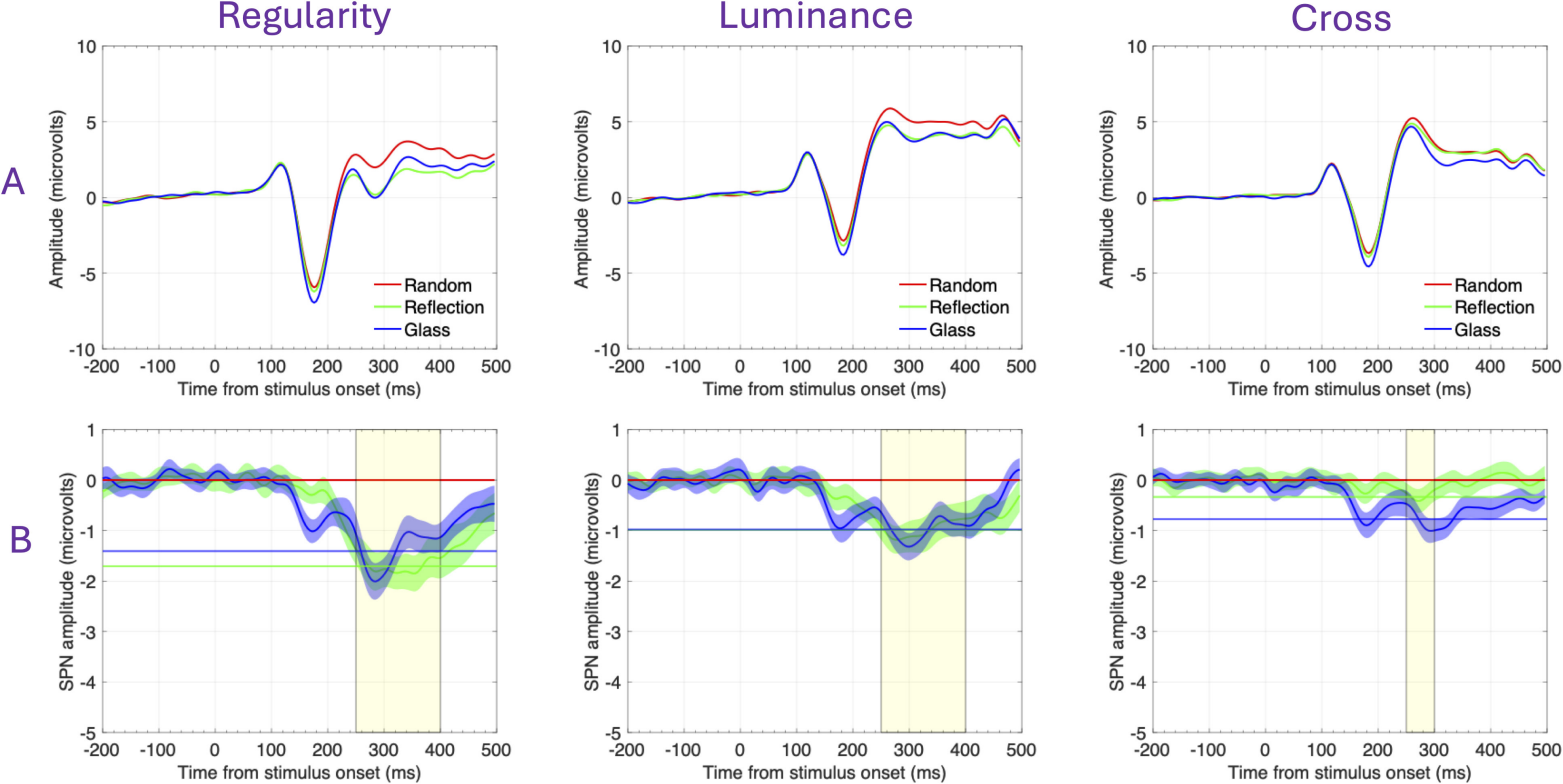
(A) Grand average ERP waves for posterior electrode cluster [PO7, O1, O2 and PO8] for all experimental conditions. (B) Grand average SPN waves with 95% CI ribbons. The vertical yellow band shows the preregistered time window (which is different in the Cross task).

**FIGURE 5:**
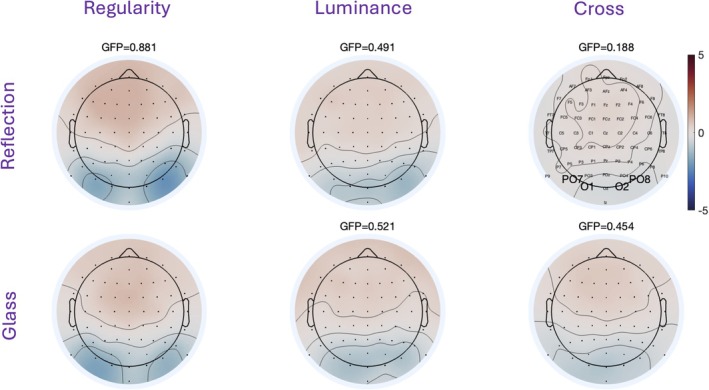
Topographic difference plots for each experimental condition.

ERP waves are shown in Figure 4, and topoplots are shown in Figure 5. The expected Task × Regularity interaction was present, *but in the opposite fashion to that which was predicted*. Contrary to predictions, Task had a large effect on reflection SPNs and a small effect on glass SPNs.

**FIGURE 6:**
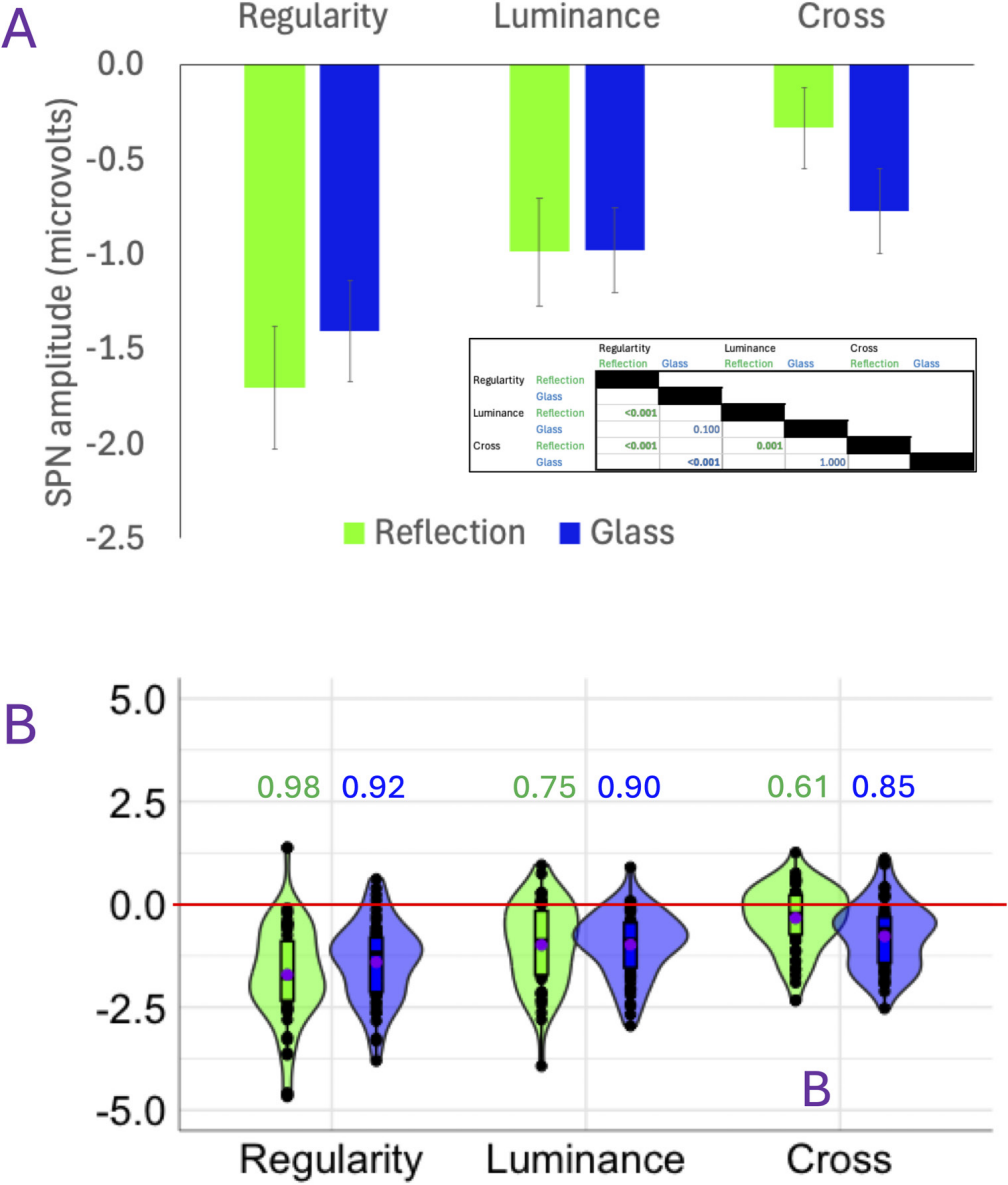
(A) Mean SPN amplitudes in microvolts for all tasks and stimuli. Error bars = 95% CI. All SPNs are significant at 0.05 level, as CI does not cross zero. *p* values from Bonferroni corrected between‐subjects pairwise comparisons are shown in the inset. (B) Violin plots showing distribution of individual participant SPNs around the means. Numbers indicated the proportion of participants where amplitude was less than zero (e.g., the SPN present). The binomial test threshold for *p* < 0.05 = 0.65, so only the reflection SPN in Cross task is nonsignificant. Note that means in Figure 6A,B are the same. These are just two different conventions for visualizing the same results.

Mean SPNs from the planned spatiotemporal clusters are shown in Figure 6A, and distributions are shown in Figure 6B. There was a significant SPN in all six experimental conditions (Regularity task, reflection *t*(51) = −10.626, *p* < 0.001, *d* = −1.474, glass *t*(51) = −10.587, *p* < 0.001, *d* = −1.468; Luminance task reflection *t*(51) = −6.964, *p* < 0.001, *d* = −0.966, glass *t*(51) = −8.901, *p* < 0.001, *d* = −1.234; Cross task reflection *t*(51) = −3.159, *p* = 0.003, *d* = −0.438, glass *t*(51) = −6.941, *p* < 0.001, *d* = −0.963). These six *t*‐tests confirm the presence of an SPN in all cases, even for Glass patterns in the Cross task, where this was not expected.

The observed differences between SPN amplitudes were confirmed with mixed ANOVA. There was a significant main effect of the between‐subjects factor task (*F*(2,153) = 22.744, *p* < 0.001, ηp^2^ = 0.229). There was no significant main effect of the within‐subjects factor stimulus type (*F*(1,153) = 0.268, *p* = 0.606, ηp^2^ = 0.002). There was a significant Task × Stimulus interaction (*F*(2,153) = 6.406, *p* = 0.002, ηp^2^ = 0.077). This is due to a larger effect of Task on reflection SPNs (*F*(2,153) = 24.775, *p* < 0.001, η^2^ = 0.245) than glass SPNs (*F*(2,153) = 7.406, *p* < 0.001, η^2^ = 0.088). Between‐subject pairwise comparisons following these main effects are shown in the inset in Figure 6A.

## 4 Discussion

Contrary to predictions, glass SPNs were less impacted by task manipulations than reflection SPNs. The reflection SPN was large in the Regularity task, reduced in the Luminance task and reduced further in the Cross task (replicating Makin et al. 2024). This was also true for the glass SPN. However, the magnitude of the task effect was smaller for glass SPNs than for reflection SPNs.

This result is surprising, as reflection has more ecological significance than glass patterns. After all, reflection is important for mate selection (Grammer and Thornhill 1994), perceptual organization (Wagemans et al. 2012), figure‐ground segregation (Driver et al. 1992) and object recognition (Treder and Meulenbroek 2010), whereas glass patterns are not important for these things.

In fact, the results suggest that the brain response to glass patterns is even more robust, preattentive and automatic than the brain response to reflection. However, this aspect of the results requires replication before it can be treated as secure knowledge.

Why are glass patterns apparently processed so readily, and perhaps even more readily than reflection? One possibility is that they resemble the visual blur caused by self‐motion. Different head movements create different shifts in the retinal image, and it is well known that this ‘optic flow’ is important for rapid visuomotor transformation (Blohm and Lefèvre 2010) and unconscious action control (Milner and Goodale 2006). Indeed, Krekelberg et al. (2005) found that glass patterns activated motion‐sensitive brain regions, and Lestou et al. (2014) found that glass patterns activated dorsal stream brain regions. The dorsal stream is usually associated with automatic and unconscious processing. Lestou et al. (2014) further suggest that a hierarchy is present in the visual cortex in that glass patterns may be detected by fast global form detectors in dorsal stream brain areas, which subsequently lead to ventral stream brain areas. This may partly explain the apparent automaticity of glass pattern processing observed by the current study.

The current study employed circular glass patterns only, whereas previous work has employed circular, radial and translational glass patterns (Rampone and Makin 2020). The current findings may not generalize to radial and translational glass patterns. Furthermore, the implied circles in the glass patterns have an infinite number of axes of reflection, including salient vertical and horizontal axes of reflection. In contrast hyperbolic glass patterns used by Wilson and Wilkinson (1998) only had cardinal axes. These hyperbolic glass patterns could be rotated to produce glass patterns with implied oblique axes of reflection only. SPN responses to oblique hyperbolic glass patterns could be unlike SPN responses to reflection.

The current study also used a single stimulus presentation duration (300 ms). While Buckley and Makin (2024) found that duration does not modulate the effect of Task on reflection SPN amplitude, it may modulate the effect of task on glass SPN amplitude. This also remains to be tested.

### 4.1 Conclusions

Contrary to predictions, the current study found that glass pattern processing was less impacted by task manipulations than reflectional symmetry processing. While this unexpected result requires replication, it seems that reflection detection is not especially preattentive, even though reflection has special ecological significance.

## Author Contributions

**Ned Buckley:** conceptualization, formal analysis, methodology, project administration, validation, visualization, writing – original draft, writing – review and editing. **Alexis D. J. Makin:** conceptualization, formal analysis, methodology, project administration, validation, visualization, writing – original draft, writing – review and editing, funding acquisition, supervision. All authors have read and agreed to the published version of the manuscript.

## Funding

This project was partially sponsored by the ESRC grant ES/S014691/1 awarded to the authors in 2019.

## Conflicts of Interest

The authors declare no conflicts of interest.

## Data Availability

All raw data, analysed data, analysis codes, stimulus images and stimulus generation codes are freely available for all users on open science framework. This is project 49 in the complete Liverpool SPN catalogue.
